# Role of retinoic receptors in lung carcinogenesis

**DOI:** 10.1186/1756-9966-27-18

**Published:** 2008-07-14

**Authors:** Krisztina Bogos, Ferenc Renyi-Vamos, Gabor Kovacs, Jozsef Tovari, Balazs Dome

**Affiliations:** 1 Tumor Biology, Budapest, Hungary; 2 Thoracic Oncology National Koranyi Institute of Pulmonology, Budapest, Hungary; 3 Department of Tumor Progression, National Institute of Oncology, Budapest, Hungary

## Abstract

Several in vitro and in vivo studies have examined the positive and negative effects of retinoids (vitamin A analogs) in premalignant and malignant lesions. Retinoids have been used as chemopreventive and anticancer agents because of their pleiotropic regulator function in cell differentiation, growth, proliferation and apoptosis through interaction with two types of nuclear receptors: retinoic acid receptors and retinoid X receptors. Recent investigations have gradually elucidated the function of retinoids and their signaling pathways and may explain the failure of earlier chemopreventive studies.

In this review we have compiled basic and recent knowledge regarding the role of retinoid receptors in lung carcinogenesis. Sensitive and appropriate biological tools are necessary for screening the risk population and monitoring the efficacy of chemoprevention. Investigation of retinoid receptors is important and may contribute to the establishment of new strategies in chemoprevention for high-risk patients and in the treatment of lung cancer.

## Background

Despite antismoking efforts and advances in therapy, lung cancer remains the leading cause of cancer-related death worldwide [1].

Multi-step carcinogenesis has been described as "a gradual accumulation of genetic and epigenetic aberrations resulting in the deregulation of cellular homeostasis" [2]. There is a similarity between bronchial lesions found in carcinogen-treated animals (squamous metaplasia) and the histological changes affecting the bronchial epithelia of humans or animals deficient in vitamin A. Restoration to a normal histological state occurs after vitamin A repletion, which in experimental models has also been shown to confer protection against pro-carcinogens.

The term retinoid (first coined by Sporn in 1976) generally refers to naturally occurring and synthetic vitamin A (retinol) metabolites and analogs [3].

Several studies have shown that vitamin A/retinoids are physiological regulators of embryonic development, vision, reproduction, bone formation, haematopoesis, differentiation, proliferation and apoptosis. Pharmacologically, they have been recognized as modulators of cell growth, differentiation and apoptosis. Furthermore they have been shown to suppress carcinogenesis in various organs (e.g. oral cancer, skin, bladder, lung, prostate and breast cancers) in experimental animals [4]. Clinically, retinoids reverse premalignant human epithelial lesions and prevent lung, liver and breast cancer and second primary tumors in the head and neck [5].

It is now generally thought that the effects of retinoids are mainly mediated by the nuclear retinoid receptors, which are members of the steroid and thyroid hormone receptor superfamily [6,7]. Two families of retinoic receptors have been identified, namely RARs and RXRs with three subtypes for each (α; β, γ) and several isoforms arising from promoter usage and alternate splicing. The retinoid receptors are ligand-activated, DNA binding trans-acting, transcription-modulating proteins. The three RAR types have a strong affinity for all-trans and 9-cis isomers of retinoic acid. The three RXR types, on the other hand, have demonstrated an especially strong specificity for only the 9-cis isomers. Studies have shown that RXR/RAR heterodimers are responsible for transducing the retinoid signal *in vivo *[8]. These heterodimers bind to retinoic acid response elements found in the promoter region of retinoic acid-inducible target genes thereby activating transcription [8,9]. Without ligand RAR-RXR, heterodimers bind to co-repressors, which play an active role in repressing the transcription of targeted genes. The recruitment of histone deacetylases (HDACs) brings about transcriptional repression by preventing the opening of chromatin, which is linked to deacetylation of nucleosomes [10]. Several of the co-activators and co-repressors are shared by multiple signaling pathways, e.g. CBP (cAMP response element binding protein) has been implicated in AP-1 (activator protein 1) and p53 signaling. Meanwhile STAT signaling, Sin3 and HDAC-1 seem to have a role in what Ayer, et. al. call "Mad-Max signaling" [4].

Dawson lists a series of nuclear receptors such as thyroid hormone receptors, vitamin D3 receptors (VDRs), peroxisome proliferator activated receptors (PPARs), and several orphan receptors in which RXR is important as a "heterodimeric partner" [11].

The RARs and RXRs exhibit the conserved module structure of nuclear receptors and their amino acid sequence can be divided into six regions (A-F) based on homology among themselves and with other members of the nuclear superfamily.

The central region C consists of 66 amino acids and has two zinc-binding motifs very much like the core of the DNA binding domain (DBD) which enables cognate response elements to be recognized specifically. Both this central C region and the functionally complex E region are highly conserved between RARs and RXRs. Region E gains its complexity from the ligand binding domain (LBD), the ligand-dependent transcriptional activation function AF-2, and a dimerization surface contained within it. The name AF-1 has been given to a second transcriptional activation function found in both the amino-terminal A/B regions.

In humans, the genes encoding RAR α, β and γ are respectively located on chromosomes 17q21.1, 3p24 and 12q13. Those for RXR α, β and γ lie on chromosomes 9q34.3, 6p21.3, and 1q22. The physiological importance of the multiple isoforms of RARs is not known precisely, but these isoforms may explain why RARs have pleiotropic biological effects.

There are two major isoforms for RARα (α1 and α2) and for RAR γ (γ1 and γ2), and four major isoforms for RARβ (β1–β4) and the recently described RARβ1' [12], whose absence seems to be responsible for retinoid resistance in lung carcinognesis. RAR isoforms can be classified as those which are transcribed from either the P1 (class I: RAR α1, β1, and β3, γ1) or P2 (class II: RARα2, β2 and β4, γ2) promoter. All class II isoform P2 promoters contain an RA response element and are RA-inducible to varying degrees [13].

Similarly, several isoforms differing from one another in their amino-terminal region have been identified for RXRα (α1, α2), RXRβ (β1 and β2), and RXRγ (γ1, γ2) [14].

### Epigenetic and genetic changes

Respiratory epithelium carcinogenesis is a multifactorial process which includes inherited and acquired genetic changes, chromosomal rearrangements, epigenetic phenomena and chemical carcinogenesis.

Vitamin A deficiency has been associated with bronchial metaplasia and increased lung cancer development. Many other factors contribute to dysfunction of retinoids and their cognate receptors [2].

The first cytogenetic reports connecting chromosome 3 to lung cancers were those of Whang-Peng et al. [15,16], who reported that 100% of small cell lung cancer (SCLC) cases examined showed specific 3p deletions by Giemsa banding. These changes were observed in 12/12 cell lines and three fresh tumors after a two-day culture period. The minimal region of common deletion was 3p14-p25. A number of studies have since been undertaken that obtained similar results that were extended to non small cell lung cancer (NSCLC).

Houle et al. mapped the RARβ2 at 3p24 and demonstrated that expression was decreased or even suppressed in lung cancer cell lines, suggesting that its re-expression could suppress malignity [17]. Frequent loss of RARβ mRNA expression has been described in both primary NSCLCs and bronchial biopsy specimens from heavy smokers [18,19]. Furthermore, in addition to lung cancer [18,20], decreased RARβ2 mRNA expression has been demonstrated in a variety of solid tumors including head and neck [21]and breast carcinomas [22]. Xu et al. [18] also reported that all RARs and RXRs were expressed in at least 89% of control normal bronchial tissue specimens from patients without a primary lung cancer and that in distant normal bronchus specimens from patients with NSCLC RARα, RXRα and γ were expressed in more than 95% of the tumor-free specimens. In contrast, RARβ, RARγ and RXRβ expression was decreased, detected in only 76% of NSCLC specimens. Picard et. al similarly showed diminished or absent RARβ protein expression in ~50% of resected NSCLCs [23]. Furthermore, these authors observed normal or elevated RARα and RXRα expression in NSCLCs. The expression of RARβ, RARγ, and RXRβ was found to be decreased, however, in many tumors, while LOH at 3p24 occurred at a high frequency. This phenomenon was also seen in non-neoplastic lesions. The authors concluded that altered retinoid receptor expression might be involved in lung carcinogenesis. Martinet et al. extended the above study investigating RARs and RXRs alteration in lung cancer precursor lesions. They performed allelotyping for microsatellites located near the RAR/RXR gene loci and immunohistochemistry was additionally carried out to evaluate P53 and RARβ expression. Microsatellite changes occurred frequently in all samples, but without specificity for any group. RARβ marker losses were found in all examined groups, with a concomitant RARβ protein expression [24].

Aberrant methylation of the promoter regions of genes is a major mechanism of gene silencing in tumors [25]. Virmany et al. [26] identified hypermethylation as the underlying mechanism for this frequent loss of RARβ expression. Twenty-one of 49 (43%) primary resected NSCLC samples showed RARβ hypermethylation. In addition, it was demonstrated that RARβ hypermethylation was also important in the pathogenesis of SCLCs, with 62% of SCLCs methylated for RARβ. In the same study, it was also demonstrated that treatment of lung cancer cell lines with the demethylation agent 5-aza-2'-deoxycytidine (5-AZA-CdR) can restore RARβ expression. Moreover, a phase I-II trial in patients with stage IV NSCLC suggests that 5-AZA-CdR may have some clinical activity against metastatic NSCLC [27]. The loss of RARβ mRNA expression has been observed in many lung cancer cell lines also suggesting that to function as a tumor suppressor gene, RARβ expression is contingent on the intracellular concentration of retinoids [28]. The effects of retinol (vitamin A) depend on its intracellular metabolism including its transport by specialized proteins such as Cellular Retinol Binding Proteins (CRBP) and on its binding as retinoic acid to specific nuclear receptors: the Retinoic Acid Receptors (RARs) and the Retinoid X Receptors (RXRs) [7]. The CRBP I and II transport retinol in the cell and serve as chaperon proteins to prevent unscheduled retinol catabolism. It is the first building block in retinoic acid synthesis.

### Retinoid signaling

The mechanisms through which retinoids suppress carcinogenesis, although complex, are gradually being elucidated. Their complexity results from the large number of genes involved in tumor cell differentiation and proliferation that include retinoic acid response elements in their promoters. Retinoids also inhibit tumorigenesis and tumor growth through their ability to induce either apoptosis (programmed cell death) or terminal differentiation. Interestingly, it has been established that the apoptotic process triggered by Retinoid Related Molecules is independent of p53 activation and proceeds through a novel pathway in which the mitochondrion seems to play a pivotal role [29].

As Karamouzis et al. stated in a recent publication [2], a 'switch on/off' model determines the relationship between retinoid receptors and other signaling pathways during bronchial carcinogenesis. According to this model, RXR selective compounds specifically inhibit AP-1 (activator protein 1) activity resulting in inhibited cell proliferation in normal respiratory epithelium, RARβ and RXRα AP-1-dependent interaction with other nuclear receptors, such as PPARγ with contribution cofactors (CBP/p300cAMP response element binding protein), ensures cyclin D1 mediated cell cycle inhibition, hence favoring apoptosis or differentiation. Down-regulation of the RARβ mechanism (as detailed above) combined with CBP and AP-1 up-regulation triggers tumor progression and proliferation. Concurrently the inability of RXRα to form heterodimers with PPARγ enables an AP1/CBP-dependent up-regulation of Cox2, resulting in the inhibition of apoptosis. This crucial role of RXRs may explain the observation of Brabender et. al as well. They observed suppressed mRNA expression of all subtypes of RXRs in curatively resected NSCLC that is followed by statistically worse overall survival [30].

In addition, retinoids play a central role in tumor stroma production and thus in the control of tumor progression and invasion through their ability to regulate the expression of matrix metalloproteinases, transforming growth factor-β, and cell cycle regulator proteins, such as cyclin dependent kinase I, such as p16, or p21 [31,32].

Up to now, the use of retinoids in clinical trials has been limited because of their pharmacologic effects and side effects. Furthermore a majority of human or experimental NSCLCs are resistant to all *trans*-retinoic acid, and the mechanism of retinoic acid resistance has not been totally elucidated. The absence of the newly recognized RARβ1' (alternatively spliced from RARβ1 isoform) could be one reason for retinoid resistance in lung carcinogenesis [12]. In that study RARβ1' expression was repressed in RA-resistant BEAS-2B-R1 cells in lung cancer, compared with adjacent normal lung tissues. In H358 lung cancer cells that were transiently transfected with RARβ1', RA treatment was able to restore target gene expression. In order to better understand the mechanism of RARβ1' repression more studies are needed, and the authors note that "potential reexpression in lung cancer may be important to future approaches to lung cancer chemoprevention" [33].

### Chemoprevention

Chemoprevention has been defined as: the application of natural or synthetic molecules to prevent, inhibit or reverse the carcinogenic machinery [34].

For the respiratory tract there are two major classes of agents which appear to prevent damage induced by inhaled carcinogens: retinoids and antioxidants. (In addition to those mentioned above, new classes of chemopreventive agents are under investigation, such as EGFR inhibitors, farnesyl transferase inhibitors, Cyclooxygenase-2 inhibitors etc., but presently we are focused on retinoids and synthetic Retinoid Related Molecules (RRMs).

Clinical trials have shown how complex the chemoprevention approach is. Nevertheless, large primary prevention trials in volunteers (physicians and nurses in the Physicians Health Study) and in high-risk populations (smokers, ex smokers and asbestos workers in the CARET and ATBC studies, and in the more recent EUROSCAN trial) using either beta-carotene, or the combination of beta-carotene and retinyl palmitate and the combination of beta-carotene and alpha-tocopherol have documented a higher incidence of lung cancer among smoking participants who received beta-carotene [35,36]. Interestingly a negative effect of beta-carotene supplementation has also been observed in experimental animals exposed to cigarette smoke [37]. In the EUROSCAN study, where retinyl palmitate and/or N-acetylcisteine supplementation were used, no beneficial effects on the incidence of second primary cancer and survival were observed. There was one exception for retinal given to workers exposed to asbestosis, which seemed almost protective against mesothelioma development [38]. One possible explanation for the failure and harm seen in the chemoprevention trials could be the procarcinogenic effect of the toxic oxidative carotene metabolites. The oxidative metabolites induce cytochrome P450 enzymes, lowering the serum levels of retinoid acid and down regulating RXR and RARβ. Nicotine by itself inhibits RARβ expression via methylation.

Further randomized, controlled chemoprevention trials designed to test retinoids, β-carotenes or α-tocopherol defined their target population based on smoking history, preneoplastic changes of the bronchial epithelium, or cancer history [39] (Table 1).

**Table 1:** Lung cancer chemoprevention trials on retinoic acid analogs

**Study**	**Drug**	**Number of patients**	**End point**	**Result**	**Reference**
**ATBC**	β-Carotene	29,133	Lung cancer	Negative/harmful	[57]
	α-Tocoferol				
**CARET**	β-Carotene	18,314	Lung cancer	Negative/harmful	[58]
	Retinol				
**EUROSCAN**	Retinyl-palmitate	2,592	Second primary tumor	Negative	[38]
	N-Acetycystein				
**Physician's Health Study**	β-Carotene	22,071	Lung cancer	Negative	[59]
**Pastorino et al.**	Retinyl-palmitate	307	Second primary tumor	Positive	[35]
**Lam et al.**	ADT	112	Bronchial dysplasia	Positive	[40]
**Kurie et al.**	Fenretinide	82	Metaplasia	Negative	[60]
**Lippman et al.**	13cRA	1,304	Second primary tumor	Negative	[61]

ATBC, α-Tocoferol, β-Carotene Cancer Prevention Study; CARET, Carotene and Retinol Efficacy Test; ADT, anethole dithioethione (5-[p-methoxyphenyl]-1,2-dithiole-3-thione; 13cRA, 13 cis retinoic acid

In a recent study Lam S. et al. observed that retinol was not effective in the up-regulation of RARβ in lesions with bronchial dysplasia among individuals who continued to smoke [40].

In addition Khurie FR. et al. reported worse prognosis in stage I. lung cancer, which indicated maintenance of RARβ expression and overexpression of RARβ correlated with increased expression of cyclooxygenase-2, an enzyme that contributes to progressive carcinogenesis and is a marker of poor prognosis [41,42].

The gene promoter hypermethylation is a leading cause of gene silencing. The loss of RARβ expression due to hypermethylation is of interest as are bronchial premalignant lesions in developed lung cancer. Recent publications show that hypermethylation of RARβ2 genes has a different effect on the development of second primary lung cancers (SPLCs) in NSCLCs depending on smoking status. In current smokers, SPLCs developed more frequently when RARβ was unmethylated than when it was hypermethylated. In the case of former smokers it was the opposite. SPLCs were more prevalent in patients with hypermethylated RARβ. Thus, in active smokers, silencing RARβ expression by hypermethylation has a protective effect against the development of SPLCs, whereas in former smokers RARβ expression (unmethylated) appears to be protective. The authors suggested that in current smokers, the continuous high oxygen tension and free radicals induce apoptosis and offer protection from the SPLCs. This apoptosis may be inhibited by retinoic acid if RARβ is expressed on a normal level [43]. These findings explain in part the previous observation that RARβ expression is associated with poor prognosis among patients who are active smokers [44].

Now available are stronger synthetic retinoids that select for an RAR and RXR type without exposing patients to the kind of retinoid toxicity that had previously been observed [45,46]. Aerosolized early on site, these retinoids have been able to reverse the RR deficiency in stabilizing RAR/RXR expression for increased ligand binding to restore normal cellular differentiation [47]. The authors, collaborating with a French research group, conceived and designed an appropriate RR assay in order to measure efficiently the normal bronchial mucosa level of each Retinoid Receptor's mRNA by real time quantitative relative RT-PCR. This method could be useful for screening the RR's status in the damaged bronchial epithelia of the high-risk patient and for monitoring the efficacy of the different Retinoids used as chemopreventive agents [48].

## Summary

There is a large body of literature on clinical and preclinical studies using natural retinoids and related compounds for the prevention and the treatment of cancer [49]. The field of lung cancer chemoprevention has been controversial until now. However, there has also been disappointment in extending the therapeutic use of bexarotene (selective RXR agonist) to patients with NSCLC. Although preclinical data and a phase II clinical trial suggested that bexarotene added to platinum based chemotherapy may improve overall survival [50], a subsequent Phase III clinical trial did not bear this out [51,52]. One possible reason is that solid tumors can acquire and develop intrinsic resistance to retinoids during carcinogenesis. The effects of receptor selective retinoids on NSCLC cell lines were examined by Sun et al. According to their findings 8 of the 37 retinoids showed growth-inhibitory activity (IC_50_, <10 μM) against at least two of the eight NSCLC cell lines [53]. CD437, a retinoid with some selectivity toward RARγ, was highly effective [54]. The RXR selective compounds did show growth inhibitory effects when combined with the RAR retinoids. These results indicated that human lung cancer cell lines have a high degree of resistance to synthetic retinoids [55]. Freemantle et al. have summarized the potential mechanisms of Retinoic Acid resistance. Increased P450 catabolism, drug export (P glycoprotein mediated), sequestration of retinoids by CRABs or other proteins, decreased expression of RARs through promoter methylation, persistent histone deacethylation, RAR rearrangement or mutation in the RAR ligand binding domain, and coactivator alteration or alterations downstream of target gene expression may lead to cellular retinoid resistance. This knowledge should aid in predicting those most likely to benefit from retinoid therapy and in developing strategies to optimize single agent or combination retinoid regimens to overcome resistance [56]. The generation of retinoids and rexinoids with restricted selectivity has opened new possibilities for cancer therapy and chemoprevention. It is probable that demethylating and chromatin remodeling agents currently under clinical investigation could be combined with these new retinoids for a better restoration of RR expression.

## Authors' contributions

KB and BD conceived of the study and co-wrote the manuscript. FR, GK and JT provided helpful comments and helped write the paper. All authors read and approved the final manuscript.
